# Evaluation of a novel therapeutic education programme for people with alcohol use disorder in France: a mixed-methods intervention study protocol (ETHER)

**DOI:** 10.1186/s12954-021-00587-0

**Published:** 2022-01-10

**Authors:** Saskia Antwerpes, Marie Costa, Marion Coste, Morgane Bureau, Gwenaelle Maradan, Christophe Cutarella, Jacques Leloutre, Olivier Riccobono-Soulier, Sophie Hedoire, Elodie Frot, Fabienne Vernier, Stéphanie Vassas-Goyard, Tangui Barré, Danielle Casanova, Patrizia Carrieri

**Affiliations:** 1 Aix Marseille Univ, INSERM, IRD, SESSTIM, Sciences Économiques & Sociales de la Santé & Traitement de l’Information Médicale, ISSPAM, 27 Bd Jean Moulin, 13385 Marseille, France; 2 Clinique Saint-Barnabé, Marseille, France; 3 CSAPA, Centre de Soins, d’accompagnement Et de Prévention en Addictologie, Association Addictions France, Digne-les-Bains, France; 4 CSAPA, Centre de Soins, d’accompagnement Et de Prévention en Addictologie, Digne-les-Bains, France; 5 CSAPA, Centre de Soins, d’accompagnement Et de Prévention en Addictologie, Association Addictions France, Avignon, France; 6 CSAPA, Centre de Soins, d’accompagnement Et de Prévention en Addictologie, Avignon, France

**Keywords:** Alcohol, Harm reduction, Patient education, Controlled drinking, Psychosocial skills, Community-based participatory research, Abstinence, Health-related quality of life

## Abstract

**Background:**

ETHER *(“Education THEérapeutique pour la Réduction des dommages en alcoologie” or Therapeutic education for alcohol-related harm reduction)* is a multicentre community-based mixed-methods study, which aims to evaluate the effectiveness of the innovative therapeutic patient education (TPE) programme ‘Choizitaconso’ in a sample of French people with alcohol use disorder (people with AUD). Choizitaconso teaches people with AUD psychosocial skills to help them (re)establish controlled drinking and reduce alcohol-related harms. Recruitment started in October 2019. We present here the protocol of the ETHER study.

**Methods:**

ETHER’s quantitative component involves a 6-month controlled intervention study which evaluates Choizitaconso’s effectiveness by comparing 30 people with AUD following the programme with a control group of 60 people with AUD not enrolled in it, using a questionnaire co-constructed by the research team and members of the people with AUD community. Thirty-four alcohol-related harms are assessed and summed to provide an individual measure of the ‘harm burden’ from consuming alcohol (primary outcome). Secondary outcomes are anticipated and internalized stigma, alcohol consumption measures, craving for alcohol, coping strategies, health-related quality of life, self-confidence to control or abstain from drinking, treatment self-regulation, anxiety and depressive symptoms, alcohol-related neuropsychological impairments, and capabilities (a measure of wellbeing in adults). Data will be collected in face-to-face and phone-based interviews at enrolment and 6 months later. Linear regression models will be used to assess the impact of the TPE programme on changes in the primary and secondary outcomes, while adjusting for other correlates and confounders. The study’s qualitative component comprises semi-structured interviews with 16 people with AUD who have already completed the TPE programme at least 6 months before the interview. Qualitative interviews will be analysed using thematic analysis.

**Results and conclusions:**

ETHER is the first evaluation study of an innovative TPE programme specifically designed to reduce alcohol-related harms and reach controlled drinking in France. The involvement of the people with AUD community in selecting which experienced and perceived alcohol-related harms to measure ensures that ETHER will provide healthcare staff and researchers with a relevant set of harm reduction criteria for use in future research. Finally, ETHER will provide scientific justification for implementing novel alcohol-related harm reduction approaches and champion controlled drinking as a therapeutic goal.

*Trial registration* ClinicalTrials.gov, NCT03954054. Registered 17 May 2019—Prospectively registered, https://clinicaltrials.gov/ct2/show/NCT03954054?cond=alcohol&cntry=FR&city=Marseille&draw=1&rank=1.

## Background

Alcohol is the second leading cause of avoidable death after tobacco. In France, 41,000 deaths—or 7% of all deaths—were attributed to alcohol consumption in 2015 [1]. From an economic perspective, alcohol was responsible for a loss to the French economy of almost four billion euros in 2010, with an estimated social cost (i.e., the total monetary and non-monetary cost because of alcohol use) of almost 120 billion euros [2]. Worldwide, alcohol is the most dangerous psychoactive substance in terms of physical and social harms for users and for society [3]. Harmful alcohol use is linked to over 200 health conditions, ranging from liver disease, road injuries and violence, to cancer, cardiovascular disease, suicide, tuberculosis and human immunodeficiency viruses (HIV) [4]. Alcohol use disorder (AUD) is a problematic pattern of alcohol use leading to clinically significant impairment or distress [5]. Its diagnosis is based on multiple occurrence of given criteria within a 12-month period, and short screening tools such as the Alcohol Use Disorders Identification Test-Concise (AUDIT-C) shows good performance to detect AUD [6]. Cognitive impairments can be found in 50–80% of people with alcohol use disorder (AUD) [7], including executive dysfunction, episodic memory deficits and visuospatial disabilities. The term ‘people with AUD’ is chosen throughout the manuscript as a non-stigmatizing, non-judgmental term based on ‘person-first language’ that shifts away from defining a person through the lens of disease [8].

There is extensive literature on evidenced-based psychosocial interventions for alcohol use disorder (AUD) [9]. Brief interventions—which comprise counselling and simple assessment of current or potential problems with substance use—are the psychosocial interventions most recommended to achieve low-risk alcohol use among hazardous and harmful drinkers [10]. Typically delivered in a single session in primary care settings, there is a great deal of evidence for their effectiveness, particularly in individuals with mild AUD and people with at-risk alcohol use who are not very concerned about their consumption [11]. Motivational interviews and cognitive behavioural therapies are two psychosocial interventions which are also effective in reducing alcohol use [12]. They are more intense than brief interventions. While difficult to quantify, psychosocial interventions may be of interest as early as a low-to-moderate AUD, while pharmacological treatments may be more suitable for moderate-to-severe AUD [9]. The examination of the combined effect of both approaches represents a great opportunity for research. There are elements pointing at the superiority of the combination over single-approach treatment [13–16].

Although AUD concerns approximately 3.5 million people in France [4], more than half of this population have never received appropriate treatment [17, 18]. In the U.S., among those with 12-month and lifetime diagnoses of AUD, only 7.7% and 19.8%, respectively, sought treatment [19]. Common barriers to seeking treatment are a lack of awareness about living with AUD*,* fear of stigmatization, and apprehension about total abstinence [20]. With regard to the latter, alternative treatment approaches have emerged in recent years that aim to minimize the harmful consequences associated with alcohol use in people who fail with, or simply refuse, total abstinence as a therapeutic option. These alternative harm reduction approaches, including controlled drinking (CD) interventions, alleviate alcohol-induced harms by reducing the total amount of alcohol consumption and by modifying drinking patterns [21, 22]. Such CD interventions have also been developed within the concept of pre-habilitation, i.e. offered to individuals prior to detoxification and while the person is still drinking [23]. A recent systematic review highlighted that “evidence does not support abstinence as the only approach in the treatment of alcohol use disorder. Controlled drinking, particularly if supported by specific psychotherapy, appears to be a viable option where an abstinence-oriented approach is not applicable” [24].

Therapeutic patient education (TPE) is a relatively recent practice, defined by the WHO in 1998 as “educational activities essential to the management of pathological conditions, managed by health care providers duly trained in the field of education, designed to help a patient (or a group of patients and their families) to manage their treatment and prevent avoidable complications, while keeping or improving their quality of life” [25]. This approach rose from the acknowledgement that citizens themselves could improve on their health through behavioral factors, and that finally, health care cannot be effective without provider–patient and provider–provider communication, as well as inclusion of the patient’s social environment in treatment decisions [26]. TPE is therefore a patient-centred, multidisciplinary approach that aims to promote autonomy, improve health-related quality of life (HRQoL) and coping skills. In France, TPE guidelines were first published by the National Authority for Health in 2007 [27].

The efficacy of TPE is growingly documented [28], as well as its cost-effectiveness [29], at least for some chronic diseases. TPE brings together several disciplines including clinical sciences, humanities and public health, using mixed (i.e., quantitative and qualitative) methods. A quantitative analysis carried out in 2009 looking at 10 years of international publications and covering 41 chronic diseases, showed a clear increase in the number of articles on TPE and a growing corpus of randomized controlled trials [30]. TPE is effective in preventing complications and in improving health outcomes, including HRQoL, for people living with obesity and other chronic diseases such as diabetes, asthma and cardiovascular disease [28]. However, its benefits in addiction medicine, especially regarding AUD, have not been explored in detail. Of the 3378 TPE programmes listed in France in January 2014, only 35 were related to substance use disorders [31].

In Canada, community-based programs aiming to reduce harms of severe alcohol use without expecting cessation of use have been implemented [32], with positive results in terms of reduction in alcohol-related harms [33]. Those programs, in addition to alcohol intervention, provided primary care services, social and cultural interventions [32]. To date, few programmes aimed at harm reduction for people with AUD have been implemented or evaluated in Europe. Two examples are Alcochoix+, a Canadian CD programme imported and adopted by Switzerland [34], and the Outpatient Group Treatment Programme for Controlled Drinking, a 10-week behavioural self-control training programme for hazardous, harmful and dependent drinkers in Germany [35, 36].

Alcochoix+ was evaluated in a Swiss cohort study of 60 middle-aged men with moderate alcohol dependence (median AUDIT score equal to 20.5 and median weekly alcohol consumption equal to 350 g) at inclusion.

At the end of the program, results showed a median weekly alcohol consumption reduction of 160 g, a decrease of the mean AUDIT score to 14.1 points, and a slight improvement in HRQoL [34]. Similar results were found in a randomized controlled trial evaluating the German CD programme in 58 middle-aged subjects (19 women and 39 men) with an average weekly alcohol consumption 560 g. By the end of the programme, individuals in the intervention group had significantly reduced their alcohol consumption by 50% (from 678 to 354 g), whereas changes in the control group were not statistically significant [35]. Therefore, more evaluations are needed in Europe to establish the suitability and effectiveness of alcohol-related TPE, especially regarding harm reduction outcomes.

The primary objective of the study presented here—entitled ETHER—is to evaluate the effectiveness of the TPE programme Choizitaconso [37] for reducing alcohol-related harms in a sample of French people with AUD using a mixed-methods approach. In France, the TPE programme Choizitaconso [37] was developed in order to reduce alcohol-related harms among adult people with AUD. In this article, we present the protocol of the mixed-methods ETHER study, which aims to evaluate this programme in a sample of French people with AUD. This detailed description of the research hypotheses, data collected and methods used may help other researchers involved in the implementation or evaluation of such TPE programmes. Analyses of data collected within the ETHER study are still ongoing, with a release of results scheduled in the coming months.

## Methods

### Description of the Choizitaconso TPE programme

The TPE programme Choizitaconso (“Make your own choice”) was developed in 2016 in Avignon by Dr. D. Casanova, in collaboration with the people with AUD community there, and then implemented for the first time in a CSAPA there [37]. Its objectives are to reduce alcohol-related harms and to improve participants’ health conditions and HRQoL by teaching psychosocial skills which help them (re)establish self-determined CD. It values the participants’ potential to control their alcohol consumption while taking into account their emotional state and their motivation to drink or abstain from alcohol, without interpreting them as being pathological. Participants are free to choose drinking goals that best fit their individual needs, in line with clinical practice recommendations for harmful alcohol use [10].

Each Choizitaconso programme session lasts for 10 weeks for each participant and consists of the following five modules, including one optional module focusing on the family environment:

1. Understanding the mechanisms that trigger and/or maintain alcohol-related difficulties.
2. Planning and evaluating personalized CD strategies.
3. Understanding and identifying external and internal influences (e.g., thoughts and emotions); identifying and managing risk situations.
4. Identifying alcohol effects and alcohol-related expectations (by developing self-observation skills).
5. Family environment: learning how to observe and evaluate familial situations in order to best position oneself and take care of oneself (e.g., learning how to better express feelings).

**Fig. 1:**
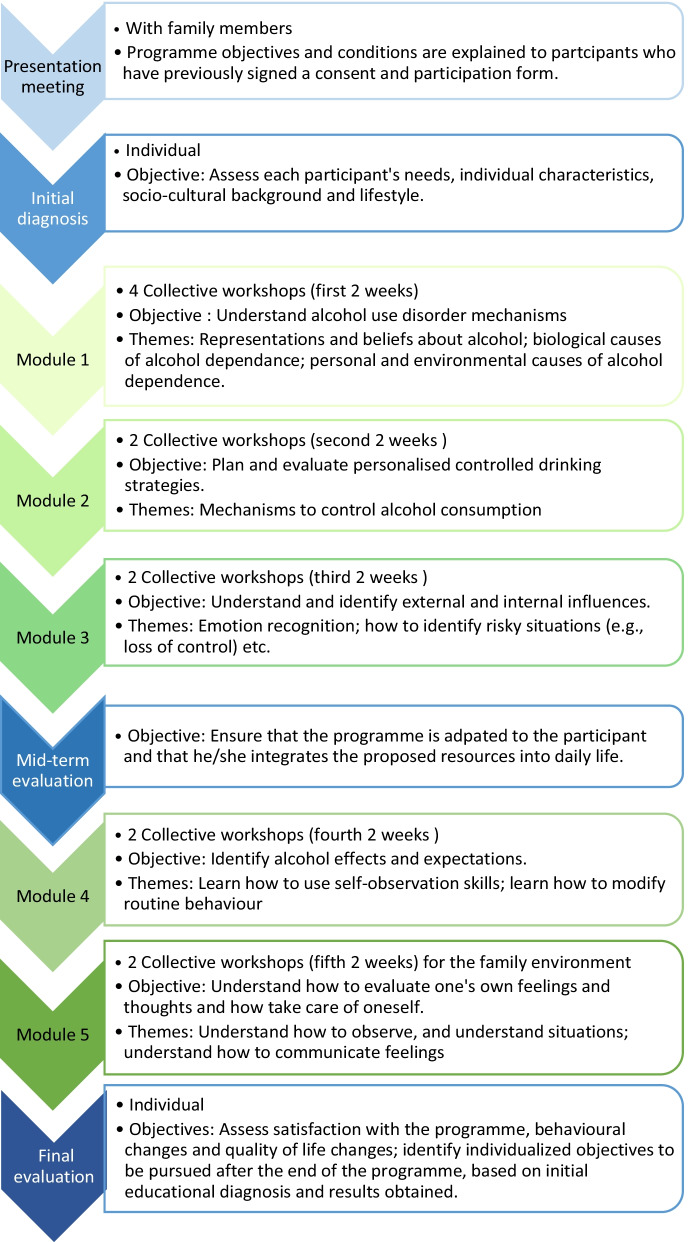
TPE programme presentation

Each module consists of two to four collective workshops that each last 120 min and involve 5–10 persons. Workshop objectives and themes are presented in Fig. 1. During the workshops, theoretical input is reduced progressively in order to promote collaborative work and co-learning between the participants.

Participants also attend several individual meetings with medical staff before, during and after the programme (cf. Fig. 1). Moreover, Choizitaconso includes remote monitoring at 3, 6 and 12 months after the programme ends, in order to assess craving, frequency and quantity of alcohol consumption and global quality of life. The multidisciplinary team in charge of the programme comprises three physicians specialized in addictology (including the programme coordinator), two registered nurses, two specialized educators, a psychologist, and a prevention manager. All healthcare providers involved in the programme have received training in TPE and substance use disorders.

#### Involvement of the people with AUD community

Peer educators (former people with AUD) collaborate in the continued development of Choizitaconso. They implemented the programme, were constantly open to participant’s feedbacks, and proposed improvements based on those feedbacks or their own experience. Unlike the healthcare providers in charge of the programme, these people with AUD are not trained in TPE but are considered experts because of their personal experience with AUD. They co-facilitate the programme’s workshops and contribute to their evaluation. If necessary, they can participate in group work during the TPE sessions and share their experiences. In addition to their collaboration in the programme’s workshops with participants, they also cooperate in research activities and discussion groups on TPE practices with the medical staff.

#### Techniques and tools used in the Choizitaconso TPE programme

Different standard TPE tools are used during the workshops including group work, brainstorming, individual work, role-plays, word lists, case studies, presentations and self-evaluation sheets.

### General study design

ETHER is an ongoing multi-centre intervention study which started in France in 2019. ETHER aims to evaluate the effectiveness of the TPE programme Choizitaconso in terms of alcohol-related harm reduction. It will provide important information for implementing and assessing this CD programme in other contexts. It will also provide healthcare professionals with an effective, validated and acceptable healthcare programme for people with AUD which can be combined, if desired, with other pharmacologic approaches according to the individual patient’s needs and wishes. It uses a mixed-methods approach which comprises (1) a qualitative study with former participants of the Choizitaconso TPE programme and (2) a controlled intervention study comparing current participants of the programme (i.e., the intervention group) with people with AUD receiving inpatient and outpatient care but not participating in the programme (i.e., the control group). This control group was included in the study design in order to check for the presence of any impairment in the outcomes for people who participated in Choizitaconso, compared with people who did not. This recurrent 10-week programme takes place in a specialized service for substance use disorders (called CSAPA in France) in Avignon. Numerous positive qualitative feedbacks from former participants encouraged the implementation of an objective evaluation in order to consider further dissemination of the programme in other CSAPAs.

Both the development of the Choizitaconso programme (which main steps are described at the beginning of the Methods section) and its evaluation (i.e. ETHER) were based on guidelines set down for community-based participatory research [38]. This approach, involving end-users in the co-creation of public health interventions is thought to increase adherence and effectiveness due to empowering end-users to develop outcomes tailored to their circumstances. ETHER included such principles by noticeably (1) collecting TPE programme users’ discourse in order to allow for benefits non reported in the quantitative study but relevant for users to emerge and be taken into account and (2) co-constructing the quantitative outcome with users in order for it to fit with their main concerns and priorities.

Written informed consent was obtained from each patient before enrolment. The protocol was undertaken in accordance with the Declaration of Helsinki and French law for biomedical research and was approved by the CPP Sud-Est 4 ethics committee (Lyon, France).

### Controlled intervention study

#### Objectives

The primary objective of the controlled intervention study is to evaluate the effectiveness of the 10-week Choizitaconso programme at reducing the number of alcohol-related harms (primary outcome) in people with AUD 6 months after programme initiation (i.e., study enrolment). The secondary objectives are to assess the TPE programme’s impact on stigma, HRQoL, alcohol consumption, craving for alcohol, alcohol-related neuropsychological impairments, anxiety and depressive symptoms, coping strategies, treatment self-regulation, self-confidence to control or abstain from drinking, and capabilities (a measure of wellbeing in adults).

#### Controlled intervention study outcomes

##### Principal outcome

The controlled intervention study comprises a 34-item ad-hoc questionnaire administered through a Computer-Assisted Telephone Interview (CATI). The items relate to 34 different psychosocial, behavioural and physical harms. They were chosen according to a Delphi approach as follows: first, a list of possible questions were identified by the study’s researchers in the international literature from existing and validated questionnaires; second, during several working sessions, peer educators (former people with AUD), approved or adapted those questions from the list which they felt would guarantee the relevance of the study outcomes. Collaboration between the peer educators and the research team followed the guidelines set down for community-based participatory research [38].

The 34 alcohol-related harms are measured as dichotomous (yes/no) variables. The controlled intervention study’s primary outcome is the number of alcohol-related harms experienced (ranging between 0 and 34). This number provides a measure of the ‘harm burden’ of alcohol use. Alcohol-related harms are assessed at enrolment for the intervention group (i.e., before the study participant commences the Choizitaconso TPE) and 6 months later (i.e., 14 weeks after the end of the programme).

For further information on the different alcohol-related harms, see Table 1 below.

**Table 1:** List of the community-validated alcohol-related harms (primary outcome)

1. Professional neglect
2. Neglecting parental responsibilities
3. Neglecting conjugal responsibilities
4. Damaging a close friendship
5. Damaging an intimate relationship
6. Damaging a family relationship
7. Impulsiveness—saying or doing something you regret afterwards
8. Driving under the influence of psychoactive substances, drugs, alcohol
9. Problems with the legal system
10. Difficulties falling asleep
11. Difficulties staying asleep
12. Waking early
13. Hot flashes and/or night sweats
14. Hangover, vomiting, being sick after drinking
15. Alcohol-related physical injury
16. Trembling hands
17. Blackouts/memory problems
18. Neglecting own health in general
19. Neglecting physical appearance
20. Neglecting hygiene
21. Taking risks (crossing the street without first looking, fighting, having unprotected sexual intercourse, etc.)
22. Not eating regularly
23. Skipping meals
24. Increased drug consumption (cannabis, cocaine, anxiolytics, etc.)
25. Financial difficulties
26. No pleasure in participating in leisure activities
27. Problems arising from alcohol consumption
28. No pleasure in the taste of wine, beer or other alcoholic drinks
29. Self-perceived social isolation
30. Little contact with family members
31. Unsatisfactory family relationships
32. Little extra-familial contact
33. Unsatisfactory extra-familial relationships
34. Finding it difficult to go and consult healthcare professionals

##### Secondary outcomes

The controlled intervention study’s secondary outcomes are anticipated and internalized stigma (stigma being of high importance as it is associated with care avoidance or delayed care [39]), quantity and frequency of alcohol consumption, craving for alcohol, coping strategies, HRQoL, self-confidence to control or abstain from drinking, treatment self-regulation, anxiety and depressive symptoms, alcohol-related neuropsychological impairments and capabilities. Each is measured at programme initiation and 6 months later. The measurement methods (face-to-face interview and CATI interview) and the questionnaires used are listed in Table 2.

**Table 2:** Study outcomes

Collection	Outcomes	Description	Outcome items
CATI interview	Number of alcohol-related harms (primary outcome)	34 physical, social and behavioural alcohol-related harms^1^ (risk taking, accidents, insomnia, etc.)	34 Community-validated items
	Stigma	Anticipated and internalized stigma	“The Substance Use Stigma Mechanism Scale” (SU-SMS) [45]
	Alcohol consumption	Frequency and quantity of alcohol consumption, binge drinking	Short form of the “Alcohol Use Disorder Identification Test” (AUDIT-C) [40]
	Craving	Craving for alcohol (strong desire or compulsion to consume alcohol)	*Community-validated items (for the three previous months)* “Did you picture alcohol or drinking?” “Did you have a strong urge to drink” “What level of control did you have over your alcohol consumption?”
	Coping strategies	Cognitive and behavioural efforts to cope with stress in everyday life (trait coping)	*“Brief COPE”* [46] Coping dimensions: (1) active coping, (2) planning, (3) using instrumental support, (4) using emotional support, (5) venting, (6) behavioural disengagement, (7) self-distraction, (8) self-blame, (9) positive reframing, (10) humour, (11) denial, (12) acceptance, (13) religion, (14) substance use
	Health-related Quality of Life (HRQoL)	Mental and physical health	*“Short-Form 12-item Health Survey (version 2)”* (SF-12v2) [47] 8 domains: physical functioning, role-physical, bodily pain, general health perceptions, vitality, social functioning, role-emotional, mental health
	Self-confidence to abstain from or control drinking	Confidence to abstain from or control drinking in high-risk situations	“Brief Situational Confidence Questionnaire” (BSCQ) [48]
	Treatment self-regulation	Reasons for starting treatment or engaging in healthy behaviour	*Adaptation of the “Treatment Questionnaire Concerning Diabetes”* [49] Two subscales: autonomous regulation and controlled regulation
Face-to-face interview	Anxiety and depressive symptoms	Self-reported psychiatric symptoms of depression or anxiety	“The Hospital Anxiety and Depression Scale” (HADS) [50]
	Alcohol-Related Neuropsychological Impairments	Verbal episodic memory, visuospatial abilities, working memory, executive functioning	“Brief Evaluation of Alcohol-Related Neuropsychological Impairment” (BEARNI) [51]
	Capabilities	A measure of wellbeing for the general adult population	“ICEpop CAPability measure for Adults” (ICECAP-A) [52] Comprises five attributes: Attachment (an ability to have love, friendship and support), stability (an ability to feel settled and secure), achievement (an ability to achieve and progress in life), enjoyment (an ability to experience enjoyment and pleasure), autonomy (an ability to be independent)

#### Recruitment and data collection

**Fig. 2:**
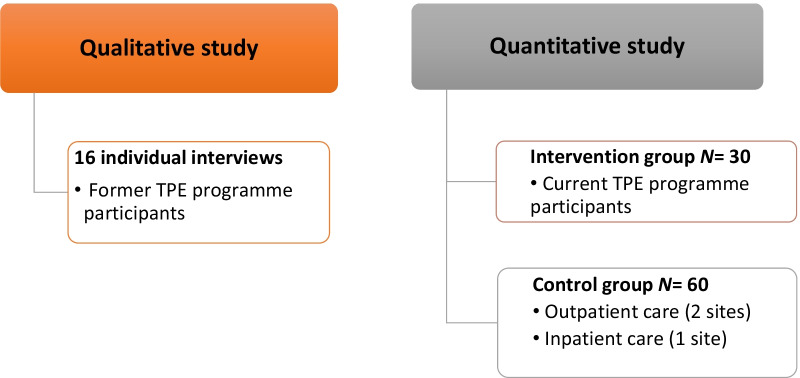
ETHER: general study design. Abbreviations: TPE: Therapeutic patient education

Patient recruitment in ETHER started in October 2019 and ended in February 2021. The principal investigator was responsible for recruiting the intervention group (i.e., participating in the TPE programme) in a CSAPA in Avignon. Four different sessions of the 10-week TPE programme enabled us to enrol a total of 34 (i.e. four more than expected) patients in the intervention group. At the beginning of a programme session, all participants were presented ETHER study and proposed to participate. Those who accepted signed the informed consent and were enrolled. The control group comprised 58 (i.e. two less than expected) patients enrolled in three sites with the support of medical staff: 30 were recruited in two CSAPA in Digne-les-Bains and in Avignon (the same CSAPA as for the intervention group but in a different service), and 30 more in an inpatient private clinic in Marseille (Fig. 2). People attending those two CSAPAs and the clinic and eligible to participate were identified by the local staff and then proposed to participate in the study during a medical or psychosocial visit. Those who accepted signed the informed consent and were enrolled. In the private clinic, participants were hospitalized for 5 weeks, and underwent a medically-supervised withdrawal. Pharmacological treatments were dispensed according to their individual needs, and participants benefited from a multidisciplinary follow-up. After the hospitalization, participants were followed-up as outpatients at the same clinic, were referred to another addictology service (such as a CSAPA), or to their general practitioner according to their geographical constraints. In both CSAPAs, the ‘treatment as usual’ consisted in tailored and patient-centred care plans. According to participant’s history, needs and demands, they regularly came to the centres to benefit from social and psychosocial follow-up and participate in individual and/or group activities. Pharmacological treatments were dispensed according to their individual needs and therapeutic objectives.

**Fig. 3:**
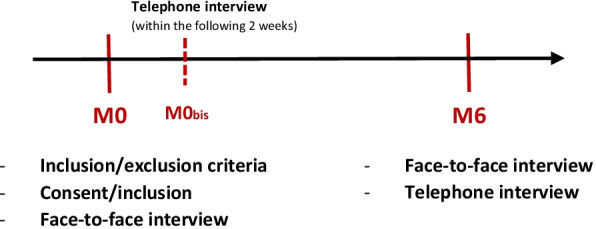
Participant timeline

Participants in both groups (i.e. intervention and control) were assessed at study enrolment (M0) and again 6 months later (M6) (i.e., 14 weeks after they end the Choizitaconso programme), using both a CATI and individual face-to-face interview (Fig. 3).

Anxiety and depressive symptoms, alcohol-related neuropsychological impairments and capabilities were assessed in the face-to-face interview. In the CATI interview, data on the primary outcome and other secondary outcomes were collected as were sociodemographic and medical data (Table 2). Alcohol consumption was measured using a short form of the Alcohol Use Disorder Identification Test (AUDIT-C) [40].

#### Sample size calculation

The CSAPA delivering the intervention could not enrol more than 25–30 persons per year. Accordingly, in order to avoid overly prolonging the study and to ensure adequate power, we decided to include twice as many controls than intervention participants. As there are no comparative estimates in the literature to predict the natural variation in the number of alcohol-related harms over follow-up after a TPE intervention, the sample size was computed according to the following assumptions: (1) the Choizitaconso TPE programme will be considered effective if it results in a reduction of at least 30% in the number of alcohol-related harms in the intervention group vs. an expected 5% reduction in the control group over a 6-month period. With an alpha = 0.05 and a power of 80%, we estimated we would need 30 individuals in the intervention group and 60 in the control group to be able to demonstrate a significant difference between both groups.

#### Inclusion criteria

ETHER’s inclusion criteria were as follows: at least 18 years old; able to provide written informed consent; fluent French speaker; reachable by phone; and followed for AUD in one of the four recruitment centres (i.e., the TPE site and the three control group sites). These inclusion criteria were consistent with the eligibility criteria for participation in Choizitaconso. Participants from the inpatient private clinic in Marseille must first complete medically-supervised withdrawal before inclusion. Exclusion criteria were cocaine or opiate dependence, pregnancy, being a legally-protected adult (tutorship, curatorship), already participating or planning to participate in another study during ETHER’s 6-month follow-up period, and having severe cognitive impairment or psychiatric disorders which could hamper an appropriate assessment of the intervention effects in the 6-month interviews.

#### Data analysis plan

The planned data analyses will be organized in several steps as follows:

(A) Descriptive analyses: sociodemographic data and alcohol consumption levels will be compared across study sites and between both the intervention and control groups at enrolment, using parametric and non-parametric tests.
(B) Comparison between enrolment (M0) and 6 months (M6) of the percentage variation in the number of alcohol-related harms, using a binomial test.
(C) Modelling of variation in the number of alcohol-related harms using linear regression, to determine whether any differences between groups persists after adjustment for possible confounders (identified in step A) or other predictors.
(D) Repeat analysis B and C for secondary outcomes using linear regression models.
(E) Post-study analysis: factor analysis to aggregate the alcohol-related harms measured at enrolment, in order to identify one or two main alcohol-related harm dimensions, and verify to what extent the intervention is effective for these dimensions.

### Qualitative study

#### Objectives

The qualitative study (commenced January 2020) consists in semi-structured interviews with people who completed the TPE programme at the CSAPA in Avignon at least 6 months before being interviewed. The TPE programme content and the clinical staff are the same for both the qualitative study and the controlled intervention study.

**The qualitative study aims to**

- Explore the link between individual factors (history of alcohol use, social and family contexts, etc.) and the decision to participate in the Choizitaconso programme.
- Observe how these individual factors influence the implementation of the alcohol harm reduction strategies taught in Choizitaconso.
- Highlight Choizitaconso’s strengths and weaknesses.

#### Sample size calculation

Sixteen to 20 semi-structured interviews were initially planned to reach data saturation. This sample size was based on the work of Ashley K. Hagaman and Dr. Amber Wutich, who found that 16 interviews or fewer are sufficient for studies conducted among homogeneous groups [41]. To date, we have performed 16 interviews and indeed, we seem to be close to data saturation.

#### Recruitment and data collection

The principal investigator first presented the qualitative study to potential participants and then invited them to participate. The contact information (including only first name and phone number) of those who agree to take part was sent by email to the qualitative study investigator who then called the participants to fix an appointment for the semi-structured interview. Interviews took place in a closed office to ensure participant confidentiality. Before performing interviews, the participant and the study coordinator signed a consent and information form. Interviews were recorded and followed the guidelines presented in Table 3. The interview guide was constructed using existing literature and with the study objectives in mind.

**Table 3:** Qualitative study interview guide

General themes	Specific themes to address
Context for entering the programme	Can you say a few words about your alcohol consumption journey? How did you get into this programme? Were you apprehensive? What were your main difficulties / the main damages related to your alcohol consumption?
Programme expectations	What did you expect from this programme? Did you have specific objectives? Which?
Assessment of TPE workshops	Did you attend all the workshops? What was your experience of the workshops? Did you have particularly pleasant or unpleasant experiences during the workshops? How did you feel at the end of the workshops? What would you say are the strengths and weaknesses of the programme? Were you able to use what you learned in your daily life, right from the start of the programme?
Feelings after the end of the programme	Did you immediately feel that these workshops were useful for you? In what way? What were you able to implement from the things you learned? Did you need help after the programme? If yes, who did you contact?
Implementation of programme learning	Today, how do you use what you learned during the programme? Can you give me examples?
Programme benefits	Today, are you able to reduce the harms associated with your alcohol consumption? How? Have you reduced the amount of alcohol you consume? In general, what impact has Choizitaconso had on your daily life? Can you give me examples?

^1^Selected (or adapted) from [53–56]

#### Inclusion criteria

Inclusion criteria were as follows: at least 18 years old; able to provide written, informed consent; fluent French speaker; completed the TPE programme at least 6 months before the interview. Exclusion criteria are the same as those for the controlled intervention study (see above).

#### Thematic analysis

Audio file transcription will be outsourced. After receiving transcripts, thematic analysis [42] will be performed. With regard to the former, four transcribed interviews will be randomly selected, read repeatedly, analysed, coded and then categorized separately by MC, (a post-doctoral researcher in public health), and SA (a psychologist and PhD student in public health). Both professionals will then discuss their respective findings together to jointly identify and decide on discourse themes. SA will then conduct the initial coding of all transcribed interviews according to the previously identified themes. After preliminary analysis, the coding framework will be discussed and approved by all the research study’s group members. SA and MC will then conduct the final coding. Results of the qualitative study will be put in perspective of the quantitative results in order to add interpretative elements to them.

## Discussion

ETHER is a controlled evaluation of a French TPE programme aiming to reduce alcohol-related harms and to improve participants’ health conditions and HRQoL by teaching psychosocial skills which help them (re)establish self-determined CD.

In the TPE literature, authors do not often describe the specific type of education intervention used or its modalities (numbers of meetings, content and duration of the intervention, etc.). This prevents future researchers and healthcare professionals from being able to reproduce these interventions and conduct implementation science. Lagger et al. [28] explored TPE programmes for chronic diseases and showed that only 4% of the 360 selected studies provided detailed programme descriptions which would allow the educative interventions to be reproduced. Instead, we strongly believe in the importance of providing a detailed description of the Choizitaconso programme specifically for this purpose. While we are aware that the level of detail provided in the present programme description does not enable for replication, main themes and methodologies implemented are provided. Through this protocol, interested researchers can be aware of what is being evaluated and get in touch with our team if interested for more details.

Choizitaconso was designed in accordance with TPE guidelines for chronic diseases [43]. It prioritizes the participants’ development of psychosocial skills and helps them to realise their full potential despite their health problems. Moreover, Choizitaconso empowers participants to develop their own needs, objectives, and healthcare decisions. In this way, it enhances participants’ autonomy to responsibly choose and adhere to their own drinking consumption goal.

In this respect, Choizitaconso represents a real alternative to approaches exclusively aimed at alcohol abstinence or at drinking reduction. This very characteristic is likely to attract more people with AUD into care, especially those who do not feel ready to or refuse to abstain from alcohol, and who need more pragmatic strategies to avoid at-risk consumption.

To our knowledge, ETHER is the first scientific evaluation of a TPE programme specifically created for people with AUD in France. Just as in clinical research, the need for evidence of a programme’s effectiveness is essential in TPE. In the present context, rigorously designed studies evaluating the effectiveness of TPE for people with AUD are needed to provide generalizable results. The research protocol presented here was specifically created for this purpose. Moreover, there is no consensus to date on the types of harms to measure in intervention studies. In this context, ETHER will provide care providers and researchers with a community-validated set of alcohol-related harms in the context of CD.

If ETHER demonstrates Choizitaconso’s effectiveness at reducing alcohol-related harms, it may influence not only AUD care practices but healthcare professionals’ and people with AUD representations about the value of CD as a therapeutic goal, in France and elsewhere. Indeed, few validated alcohol-related TPE are implemented in Europe, and the importation of foreign interventions does not necessarily fit the cultural context around alcohol consumption and users’ needs. Establishing the harm-reducing effectiveness of this programme may lead to an in-depth description of this latter in a (scientific or not) support that can be disseminated and enable replication. It may also lead to explore the cost-effectiveness of the programme in the future. The burden associated with alcohol use is so high that innovative non-pharmacological interventions like this should be evaluated “per se” but also as strategies to be included in a comprehensive model of care including pharmacologic treatments.

Combining results using a qualitative and quantitative mixed-methods approach, ETHER will provide a more extensive and in-depth view of patients’ perceptions and experiences of the Choizitaconso TPE programme than that offered by separate analyses. More specifically, the qualitative component will help us explore patients’ perceptions long after completing the TPE programme, and will provide information which may be missed in the quantitative study. For instance, former users may be able to clarify what has been the most useful part of the programme for them, or explicit the relationship between parts of the programme and some ETHER’s quantitative outcomes. Such valuable results may thus participate in a better understanding of mechanisms involved in the harm reduction process, as well as potentially provide some insight for improving the intervention.

## Limitations

Our study has limitations. First, assignment to the two study groups (i.e., intervention and control) is not randomised. Nevertheless, specific statistical methodology (selection models) can be applied later, if needed, to control for non-randomization bias. Second, we are using self-reported alcohol use measures, which may be subject to social desirability bias. However, the validity of self-reported alcohol use has been proven in previous research [44], especially when trained interviewers administer the interview in a non-judgmental environment. Lastly, our primary outcome, tailored to our participants, is not yet validated, but we aim to check for its internal consistency during analyses of the results using Cronbach’s alpha.

## Conclusion

ETHER’s results will pave the way for the possibility of far-reaching developments, including the adaptation and implementation of the Choizitaconso TPE programme throughout Europe, with CD as a therapeutic goal. The programme might also be adapted to other substance use disorders (e.g., cannabis, tobacco) or non-substance addictive behaviours (e.g., pathological gambling, binge eating). Implementing CD programmes in substance use disorder treatment may enable users to control their alcohol intake and to better focus on harm reduction strategies. Choosing one’s own therapeutic goal—thereby becoming an actor in one’s own healthcare management—is likely to be effective for people suffering from substance use disorders other than AUD, and this needs to be studied in greater detail in the future. Moreover, this type of intervention could be offered in low-threshold centres (i.e., harm reduction centres) for drug users, and be made available to populations with psychiatric comorbidities.

### Trial status

Enrolment in this trial ended in February 2021. Data collection is ongoing at the time of resubmission.

## Data Availability

The datasets used and analysed during the current study will be available from the corresponding author on reasonable request.

## Abbreviations

AUD: Alcohol use disorder
CATI: Computer-assisted telephone interview
CD: Controlled drinking
CSAPA: *Centres de Soins, d'Accompagnement et de Prévention en Addictologie*
ETHER: *Education THEérapeutique pour la Réduction des dommages en alcoologie*
HIV: Human immunodeficiency virus
HRQoL: Health-related quality of life
TPE: Therapeutic patient education
